# Supplementary figures and images for: The Multiomics Response of Bacillus subtilis to Simultaneous Genetic and Environmental Perturbations
Source: Microorganisms. 2023 Jul 30;11(8):1949. doi: 10.3390/microorganisms11081949 (PMC10458161; doi:10.3390/microorganisms11081949)

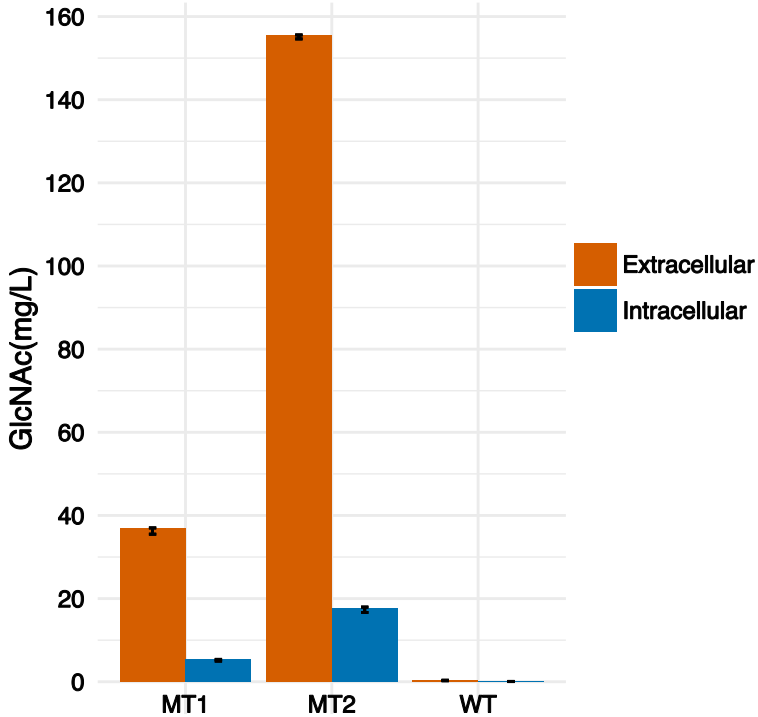

Supplement: Supplementary file 1 [file microorganisms-11-01949-s001.zip › Figure_S1.pdf]
